# Cellulose for the Production of Air-Filtering Systems: A Critical Review

**DOI:** 10.3390/ma15030976

**Published:** 2022-01-27

**Authors:** Martina Lippi, Laura Riva, Manfredi Caruso, Carlo Punta

**Affiliations:** Department of Chemistry, Materials, and Chemical Engineering “G. Natta” and INSTM Local Unit, Politecnico di Milano, 20131 Milano, Italy; martina.lippi@polimi.it (M.L.); laura2.riva@polimi.it (L.R.); manfredi.caruso@polimi.it (M.C.)

**Keywords:** filtering systems, ultrafiltration, mechanical filters, chemical filters, cellulose, cellulose-based systems, cellulose-reinforced systems, nanocellulose

## Abstract

The control of airborne contaminants is of great interest in improving air quality, which has deteriorated more and more in recent years due to strong industrial growth. In the last decades, cellulose has been largely proposed as suitable feedstock to build up eco-friendly materials for a wide range of applications. Herein, the issue regarding the use of cellulose to develop air-filtering systems is addressed. The review covers different cellulose-based solutions, ranging from aerogels and foams to membranes and films, and to composites, considering either particulate filtration (PM_10_, PM_2.5_, and PM_0.3_) or gas and water permeation. The proposed solutions were evaluated on the bases of their quality factor (QF), whose high value (at least of 0.01 Pa^−1^ referred to commercial HEPA (high-efficiency particulate air) filters) guarantees the best compromise between high filtration efficiency (>99%) and low pressure drop (<1 kPa/g). To face this aspect, we first analyzed the different morphological aspects which can improve the final filtration performance, outlining the importance on using nanofibers not only to increase surface area and to modulate porosity in final solutions, but also as reinforcement of filters made of different materials. Besides the description of technological approaches to improve the mechanical filtration, selected examples show the importance of the chemical interaction, promoted by the introduction of active functional groups on cellulose (nano)fibers backbone, to improve filtration efficiency without reducing filter porosity.

## 1. Introduction

**Figure 1:**
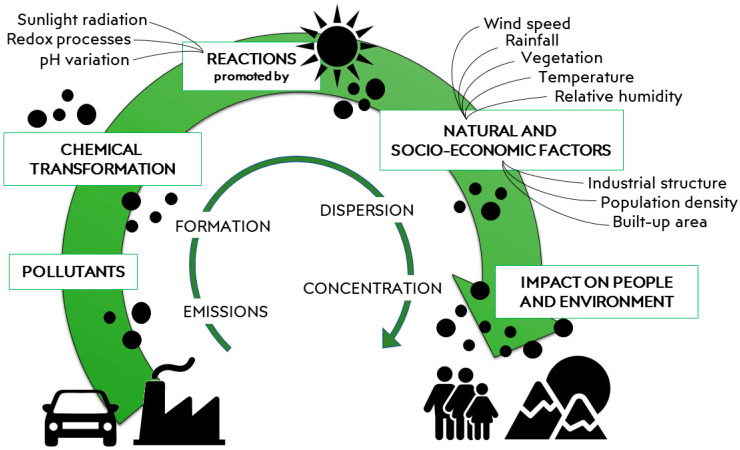
Cycle of pollution from the emission to the impact on people and environment.

Air pollution has increased globally as a result of rapid industrial growth [1]. Several aspects affect the distribution of contaminants in the air and soil, namely natural, chemical, and socio-economic factors [2]. Natural factors include rainfall, wind speed, temperature, and presence of vegetation. The chemical ones act on the decomposition of contaminants (i.e., by sunlight induced photo-degradation) and/or the kinetics of their release, mainly due to the pH variation of the environmental medium (water and soil). Finally, among socio-economic factors the most impacting aspects are certainly the build-up area, the population density, and the industrial structure of the territory (Figure 1) [2].

The search for solutions to reduce air pollution has long been a matter of interest as it is currently known how it is a high health risk factor, even greater than cigarette smoking [3,4].

**Figure 2:**
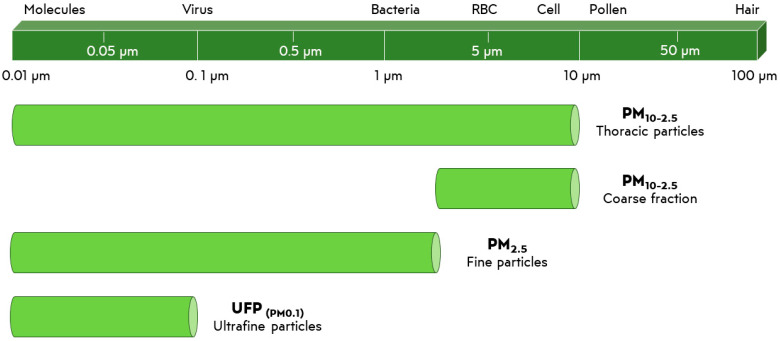
Characterization of particulate matter (PM) based on size. RBC = Red blood cell.

Air pollution aerosol particles are typically composed by microscopic solid or liquid substances generally classified as a particulate matter under 10 (PM_10_) or 2.5 μm (PM_2.5_) [5,6] (Figure 2).

Research on filter membrane materials for these particle sizes has been widely conducted, and commercially available products have recently emerged [7]. However, for particles smaller than 0.3 μm, proper filtering systems are still under development.

Currently, due to increased environmental awareness, the challenge is to achieve systems that solve problems such as environmental pollution through the design of green products and sustainable processes. The primary ambition of sustainability, which by definition aims to meet the needs of the present generation without compromising the ability of future ones to meet their own needs [8], is to create a material from the environment for the environment.

In this perspective, this contribution aims to overview recent contributions on the development of cellulose-based filtering materials, focusing on the aspects of air pollution removal and gas permeability, while antimicrobial and antibacterial properties will not be herein discussed.

Cellulose is the most abundant polysaccharide on Earth, which can be obtained from a wide number of sources, e.g., cell walls of wood and plants and some species of bacteria and algae [9].

Limiting our overview to the results reported in the literature mainly in the last decade, in the following, after a brief introduction to the main filtration properties which should be sought, we critically analyze several cellulose-based and cellulose-reinforced systems with characteristics suitable for ultrafiltration, mainly focusing on the difference in chemical and mechanical barrier mechanisms. A particular attention is paid to those structural features which control all the parameters that define the quality of a proper gas and particulate filtration system.

Cellulose-based filters and composites have also found ample application in the field of CO_2_ capture and storage. For this topic, which would require an ad hoc discussion and for this reason is not object of this contribution, we refer the reader to recent and specific review literature [10].

## 2. Filtering Systems: An Overview

### 2.1. Properties

Manufacturing of air filters requires the consideration of several aspects. A high-quality filter must be characterized by a low pressure drop, consisting of a high permeability and a large surface area which should favor the capture of more particles [11,12].

Other important parameters that characterize filters are the filtration efficiency and the quality factor. Pressure drop (Δ*P*) can be determined from Equation (1) [13] and it consists in the difference between upstream and downstream pressures. Filtration efficiency (η) can be calculated by Equation (2) [13,14], on the bases of the particles’ concentrations, C (particles/m^3^), measured upstream and downstream of the filter. Efficiency and pressure drop are often combined into a single parameter called the “quality factor” (QF), determined by means of Equation (3) [13,14], where n is the number of layers of the same filter [15]. A high value of filtration efficiency and a low value of pressure drop combine to give a filter with a high quality factor, which is with values higher than 0.01 Pa^−1^ [14,16].

(1) $$∆P=P_{upstream\;}-P_{downstream}$$

(2) $$\eta =1-\frac{C_{downstream}}{C_{upstream}}$$

(3) $$QF=\frac{-ln{\left(1-\eta \right)}^{n}}{n∆P}=\frac{-ln\left(1-\eta \right)}{∆P}\;$$

Fibrous materials are currently among the most commonly proposed for the air-filtration, as they are able to capture aerosol particles from gas stream by their fibers though Brownian diffusion, direct interception, inertia impact, gravity setting, and electrostatic deposition. These mechanisms control the air filtration process and are strictly related to the airflow velocity, the particle properties, and the morphology of the air filtering materials. Therefore, it must be evaluated that the quality factor increases once the morphological properties of the air filtering materials (AFMs) are in accordance with the airflow velocity and the particle properties, to guarantee a good balance between the filtration efficiency and the pressure drop. For fibrous filters, thickness, and solid volume fraction, together with the increase of the porosity, can be beneficial to reduce pressure drop and, at the same time, thinner fibers as well as their interconnectivity and the larger specific surface area contribute to high filtration efficiency. Moreover, the electrostatic and chemical interaction between the filter and the particulate can also significantly affect the filtration performance [14,17]. Recently also the efficiency of electrospun fibers in air filtration was summarized by Lu and coworkers, emphasizing all the morphological properties that allow the air filtration efficiency to be improved [18].

### 2.2. Filtration Mechanisms

#### 2.2.1. Mechanical Filtration

As described in the previous section, mechanical filtration mechanisms can be depicted as a function of dimensionless numbers determined by structural parameters [19]. Thus, the control and optimization of these structural properties provide a pathway to design high-performance air filters. For example, fiber diameter is a primary design factor in air filters. In particular, nanofibers, which are fibers characterized by at least one sub-micron dimension, are highly desirable due to their advantages over microfibers in a variety of aspects including filtration efficiency, more penetrating particle size, and slip effects [11,12,20,21].

Mechanical filtration filters include air filters with modified fiber morphologies [14]. Larger surface areas are able to promote particle capturing by Brownian diffusion and the reduction of the fiber diameter leads to greater specific surface areas [11]. The modification of the morphology of the fiber surface can be considered an alternative method to increase the exposed surface area, thus improving filter performance. The introduction of nanoparticles on the surface of the filters has the advantage of making the fibers rough, leading to a reduction in pressure drop by allowing additional flow lines around the fibers [22,23], and to an increase in frictional forces between the particles and the fibers, promoting efficient PM capture [24].

The introduction of hybrid structures can be also considered as a way to improve filter performance, since the use of homogeneous materials can be disadvantageous in some cases, especially in terms of permeability [11]. Hybrid structures containing fibers/components with different sizes or dimensions may offer a route to balance filtration efficiency and air permeability. A variety of such hybrid structures with multimodal diameters [25,26], dimensions [26,27], and nanonet/scaffold dual networks [28,29] have been proposed. Cellulose-based hybrid systems will be discussed in detail in the following sections.

Another method of enhancing filtration efficiency is the increase of the thickness and weight of the air filter, which results in a larger filter area and a longer retention time of particles inside the filter [19]. The effect of increased thickness can be achieved through longer fiber spinning times in one-step processes. However, this production method leads to filters with non-optimal structural properties, in particular with an excessively dense network and extremely small pores [11]. The direct consequence is large pressure drop and short operating life due to frequent clogging of the narrow pores by larger particles. These problems associated with the one-step manufacturing process have led to a different manufacturing technique by stacking multiple filter layers into a single filter unit, which allows desirable packing density and pore size while increasing filter thickness and weight, thus achieving better QF and longer life.

Not less important, it has been reported how also the moisture effect should be considered to better evaluate the final filtration performance [30].

#### 2.2.2. Chemical Filtration

Mechanical filtration approaches have limited possibilities of improvement, as a higher filtration efficiency is inevitably associated with a high flow resistance. For this reason, alternative mechanisms to mechanical filtration have been evaluated. Among these, the chemical filtration approach can be considered as a complementary solution that imparts better qualities to the filter and improves its efficiency by limiting the increase of pressure drop. Following this approach, it is crucial that both the filters and the filtration media possess the chemical features suitable for their chemical interaction. The chemisorption mechanism can occur by a cation exchange reaction, or the formation of hydrogen bonds and Van der Walls interactions, up to the formation of real covalent bonds [31]. Therefore, a specific design of the filtration material must be done according to the chemical feature of the target solute. The efficiency of many filtration systems utilized for water purification from metal ions, dyes, or for CO_2_ capture is closely related to the formation of weak chemical interactions.

## 3. Cellulosic Materials as Air filtering Systems

### 3.1. Cellulose and Nanocellulose-Based Materials

Bio-based fiber materials with good mechanical strength and gas permeability are desirable for developing novel environmentally friendly filtering systems. In this terms, cellulose as the most abundant polysaccharide present in nature can asset numerous chemical and physical properties [32,33]. In particular, the renewable nature, biodegradability, morphological features, and the chemical versatility are all qualities which make cellulose an excellent matrix for the production of composites with a wide range of applications [32,34]. In addition, by extracting cellulose at nanoscale [35,36], the defects related to the processability of cellulose composite materials, mostly due to the low dispersibility of this polymers in many solvents, can be overcome [37].

**Figure 3:**
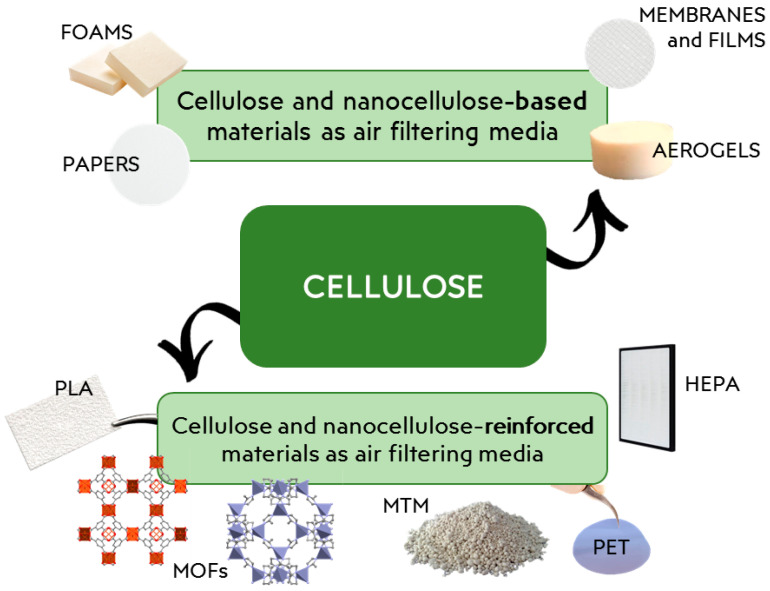
Different filtering systems obtainable from cellulose, discussed in detail in Section 3.1 and Section 3.2.

Due to its remarkable mechanical and thermal properties and its enhanced surface area, nanocellulose has been employed as rigid and solid building block of novel environmentally compatible composites in form of solids, films, aerogels, and foams [38,39,40,41,42] (Figure 3).

Barrier properties as well as filtration performances can be successfully sustained by their network structure formed by fibers that in some cases flow in low-density open porous frameworks [43].

Herein an admittedly personal selection of cellulose and nanocellulose-based materials described as potential filters for volatile molecules is reviewed, in order to highlight the progress in the adoption of these sustainable and eco-friendly materials for applications in different crucial sectors, such as food packaging and environmental decontamination. Table 1 summarizes the main aspects of the filtering systems herein discussed.

#### 3.1.1. Aerogels

**Figure 4:**
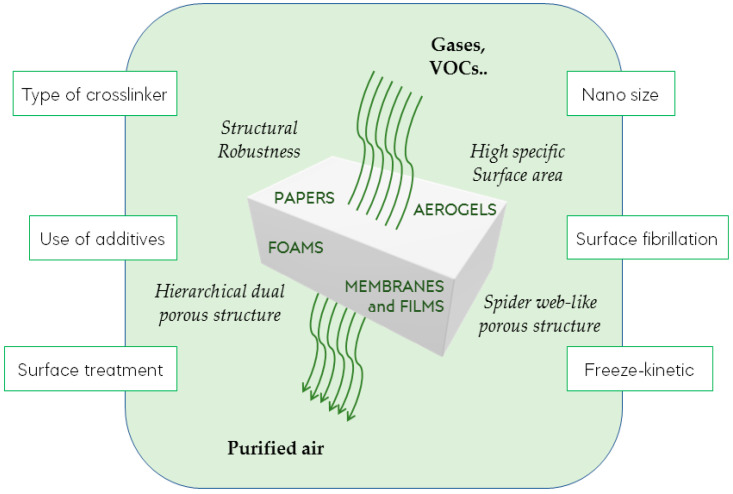
Summary depiction of the ideal characteristics that provide good filtering properties to a system, and of the general methodologies to achieve these features.

Combining the sustainability, biocompatibility, and biodegradability of cellulose to the lightweight open porous structures of aerogel systems is a great advantage for the production of promising materials [44]. Currently, research on cellulose and nanocellulose-based aerogels has gained large interest [45,46,47], and our research group has also widely contributed to the development of these porous materials by promoting the cross-linking of cellulose nanofibers for different purposes, including water decontamination [48,49,50,51,52], sensing [53,54], controlled drug-release [55], and more recently also for heterogeneous catalysis [56,57]. Made from gels, which result in interconnected nanostructures by converting the liquid phase in gas commonly though a freeze-drying process, aerogel systems present a large surface area and a low density [58,59]. These features may represent key values for producing efficient air filtration systems, whose performance in this specific application area can be sustained by further improving the structural robustness, the physical, and the chemical features of the aerogel. The most common strategy is that of creating polymer composites by carefully selecting specific additives and/or cross-linkers to develop specific properties in the final material [60,61] (Figure 4). Fan et al. reported lightweight cellulose-based aerogels with low-density and mesoporous structures, which showed increased mechanical properties and excellent hydrophobicity after crosslinking with glutaraldehyde and additional surface treatment with trimethylchlorosilane [62]. These aerogels were first presented as novel promising candidates for the adsorption of organic solvents such as nitrobenzene, benzene, chloroform, and later, their filtration capacity, comparable to high efficiency particulate air (HEPA) membranes for lampblack, was highlighted [44]. Meanwhile, their morphological structures provide low values of pressure drop as a good indicator of promising filter materials.

The regulation of the porosity of a fiber structure is also important to control the final filtration efficiency. As far as cellulose-based aerogel systems are concerned, the morphology of the porous network can strongly depend on the homogeneity of cellulose aqueous dispersion, the freezing kinetics, and the type of freeze-drying medium [46,63]. (2,2,6,6-Tetramethylpiperidin-1-yl)oxyl (TEMPO)-oxidation or enzymatic hydrolysis can be exploited as chemical pretreatment to regulate these parameters [64]. Kwang-Soo et al. synthesized different microporous cellulose filters by oxidation or enzymatic pretreatment of paper mulberry pulp in order to produce suitable material for the indoor dust removal [65]. Indeed, they evaluated the filtration efficiency of the papers as a function of their physical properties mostly induced by their synthetic processing. The major benefit in removal of fine dust (average PM_10_) was observed when water was exchanged with *t*-butyl alcohol (TBA) prior the freeze-drying process, leading to a porous network with an average pore size of 7 μm. On the contrary, chemical and biological pretreated materials showed networks with large pores, which only after 40 and 80 min respectively reached a removal efficiency of 99% against the 20 min of the TBA-based filter without any excessive increase of the pressure drop value.

The use of TBA in processing TEMPO-oxidized nanocellulose aerogels as air filters was also reported by Isogai and co-workers [13]. In this study, TBA was simply added to the TEMPO-oxidized cellulose nanofibers (TOCNF)/water dispersion leading to the formation of a spider-web-like network with a high specific surface area. The scope of this work was to combine the TOCNF-aerogel with ultra low penetration air (ULPA) and HEPA filters in order to increase their filtration performance. While the pressure drop value remained in the same range, the filtration efficiency increased from 0.0256 to 0.0357 and from 0.0273 to 0.0383 for particles with average size of 0.125 and 0.175 μm, respectively, mainly as a consequence of the morphological features of the TOCNF-aerogel.

Recently, the gas permeability of aerogel systems with hierarchical mesoporous networks was reported by Ganesan et al. [66]. As novel synthetic process, they proposed an emulsion oil template method to form a hierarchical dual-pore aerogel, whose gas permeability of the atmospheric air was carefully evaluated. In particular, the authors described the influence of the physico-chemical properties of the surfactant template on the final aerogel structures. For example, it was found that a different hydrophilic–hydrophobic balance of the surfactant can result in a different specific surface area and pore size, thus influencing the filtration activity. According to the Carman–Kozeny model [67], the secondary porous structures increased the gas permeability of the aerogel up to 178 times. In 2008, Mao et al. described freeze-dried filters made from surface fibrillated wood pulp which closely reproduced the air-filtration efficiency and pressure drop value of N95 (according to Occupational Safety and Health classification) respiratory devices [68]. They proposed a partial freeze-drying treatment of the material in order to retain the fibrillated network and thus their high specific surface area, guaranteeing their air filtration properties. With the same ambition for the same materials, later Macfarlane and co-workers [69] evaluated the role of pulp type, freezing kinetic, and the porous network in affecting the final filtration performance. Freezing the formulation in a cold plate afforded to filters displaying two layers of a high and low permeability resulted from a located structural compression. Interestingly, it was found that the addition of anionic polyacrylamide in the formulation prevents pores compression, thus increasing the effectiveness of the filters. Moreover, the impregnation of cellulose filters with activated carbon was proposed as a good strategy to gain increased VOC removal ability. The example reported by Kim et al. refers to an activated carbon cellulose filter with adsorption capacity for benzene, *m*-xylene, toluene, and methylbenzene [70].

#### 3.1.2. Membranes and Films

The research on innovative bio-based membranes and films has been extremely supported by the constant need to improve the efficiency of materials for many application fields such as food packaging [71], electronic devises [72,73,74], gas adsorption and separation [75], purification [76,77], and drug delivery [78,79].

Currently, the exploitation of polymeric materials with specific chemical and physical properties to develop stimuli responsive membranes or films, is the object of many researches [80]. The reactive properties of cellulose can be indeed a good advantage to produce membrane or films with specific characteristics. In particular, for adsorption applications, also the high surface area of nanofiber membranes can be extremely useful. Therefore, nanosized cellulose has gained an important role in developing novel membranes with good gas permeability. In particular, layered films consisting of cellulose nanofibers have demonstrated to be a great deal as oxygen barrier.

Isogai and co-workers have reported in some works the oxygen-barrier properties of TOCNF self-standing films [81], investigating the gas permeability of hydrogen, carbon dioxide, and nitrogen for the TOCN-COONa and TOCN-COOH layers [82,83]. Gas permeability tests firstly showed that the kinetic diameter of gas molecules is inversely correlated to the adsorption capacity of both layers. This explained that the gas permeates through the layers by a diffusion mechanism. Additionally, they also demonstrated a selective permeation of H_2_ over O_2_, N_2_, and CO_2_, explaining how the diffusion of the gas is largely controlled by the size of the free spaces present between the nanofibrils of the TOCN layers.

In another work, the same authors fabricated filtering films from highly purified cotton linters pulp [84]. Alkali/urea treatment and subsequent surface modification of this material with cationic alkyl ketene dimer (AKD) yielded transparent and water-repellent films. Apart from increasing the overall hydrophobic properties of the material, the introduction of lipophilic carbon chains in the structure improved the oxygen-barrier properties of the films under high relative humidity conditions. Indeed, the oxygen permeability of the filters decreased from 0.56 and 5.8 to 0.13 and 2.1 mL μmm^−2^ day^−1^ kPa^−1^ at 50 and 75% relative humidity passing from a 0 to a 0.2% of AKD content. Finally, the treatment of the films with AKD was found to be beneficial also for the improvement of their mechanical properties such as tensile strength, Young’s modulus, and work of fracture, revealing the high potential of surface modification techniques.

Membranes consisting of bacterial cellulose alone and combined with magnetite nanoparticles demonstrated to be efficient filtering systems for polar and non-polar VOCs [85]. Simulating the experimental conditions, a mathematical model was developed in order to estimate the adsorption dynamic. Both the measured and simulated results revealed enhanced adsorption performance and faster kinetics once the temperature increased, suggesting a chemo-mediate adsorption process.

Fan et al. proposed a composite material obtained by microcellulose fibers from wood pulp reinforced by protein-functionalized bacterial nanocellulose. This hierarchical structure showed the morphological features needed to well preserve a good balance between the filtration efficiency of above 99.5% for PM_1−2.5_ and the low value of pressure drop of 0.194 kPa/g. The functionalized nanocellulose showed nanoprotrusion, which facilitates the aerosol particle capture, maintaining a reduced air flow resistance due to the large pores formed by the long nanofibers [86].

Using a one-step electrospinning method, Bargis et al. instead synthesized cellulose-polyvinylpyrrolidone (PVP) fibers characterized by nano- and submicron-size. This was feasible by controlling the thixotropy and high zeta potential of the TEMPO-oxidized cellulose fibers as well as by tuning the experimental conditions. Once combined with an HEPA filter, the resulting dual-size cellulose-PVP composite increased the QF of the commercial filter about 10 times more (0.117 Pa^−1^) This high ability of collecting air particulate was attributed to its mixed sized morphological structure [87].

#### 3.1.3. Papers

The use of renewable and bio-based raw materials is becoming more and more consolidated in the paper industry. The use of cellulose nanofibers (CNF) is a great advantage for producing filter papers with strong mechanical and adsorption properties. However, the nanoscale dimensions and the hydrophilicity of CNF make their processing longer and more expensive. Wågberg and his co-workers proposed a method consisting of TEMPO and periodate oxidation treatments delaying the nanofibrillation of the fibers until after the sheet was formed [88]. In particular, an in situ pH-induced self-fibrillation method was described for producing transparent and gas barrier nanopapers with good mechanical properties, by exploiting a conventional papermaking process. The oxygen permeability (0.69 cm^3^ μm m^−2^ d^−1^ kPa^−1^ at 50% RH) resulted comparable to that of nanopapers of CNF obtained by the traditional and longer synthetic process. High filtration efficiency can be achieved also in the case of microfibrillated cellulose, as reported by Zhang and co-workers [89]. In this work, microfibrillated cellulose fibers from softwood bleached kraft pulp, were dispersed in a water/TBA mixture prior to the freeze-drying step. As in the examples mentioned above, the use of TBA induces morphological changes of filters, moving from a lamellar to a spider web-like structure, improving their filtration performance. The filtration efficiency against PM_0.3_ increased from 59 to 99% moving from a 0 to a 10% content of TBA, with a limited increase of the pressure drop of ca. 50 Pa. Further addition of TBA in the filters resulted to be deleterious for the pressure drop, which further raised from 50 to 400 Pa.

#### 3.1.4. Foams

The introduction of aqueous foam medium during paper-making allows energy saving due to the reduction of drying time [90]. At the same time, foams are characterized by a 3D structure, which prevents fiber flocculation, contributing to the formation of lightweight papers with open porous structures. In this way, the final material exhibits advantageous structural features for promising applications. Jahangiri et al. produced foams-papers from different natural sources (northern bleached softwood craft (NBSK), chemical thermo-mechanical pulp (CTMP), and nanofibrillated lyocell fiber (NLC)) with various weight ratios, in order to make filters with acceptable pressure drop values and high filtration efficiency [91]. The produced samples showed different air-content foams depending on the drying method applied. All the materials showed high air-content permeability because of the large channels formed among the fibers. However, this factor negatively affected the filtration properties, although paper foams of nanofibrillated fibers with high specific surface area could ensure submicron filtration performances even at high air-content. Moreover, Valley Beaten (VB) and Hardwood (HW) cellulose were included as additives. In particular, the addition of VB cellulose to NBSK with higher ratio made possible the obtainment of a dramatic increase of filtration efficiency.

Very recently, Ukkola and co-workers reported a new class of highly porous nano-structured foams, derived from the freeze-drying of cellulose nanofibers hydrogels in the presence of two silane compounds (methyltrimethoxysilane and hexadecyltrimethoxysilane), whose role was of both strengthening the structure by promoting the cross-linking of nanofibers, and enhancing the hydrophobicity of the nanofoams [92]. The researcher reached the best QF compromise starting from a 0.3 wt% nanocellulose hydrogel, while higher concentrations (0.7 wt%) provided very high filtration efficiencies, but high pressure-drop values as well.

### 3.2. Cellulose and Nanocellulose-Reinforced Materials as Air Filtering Media

As previously remarked, the use of homogeneous filters has several disadvantages, especially in terms of pressure drop with a negative impact on the quality factor [11]. The use of hybrid systems, which combine the properties of different materials, can be considered a smart way to enhance filtration efficiency. Thus, cellulose nanofibers, with their large exposed surface area, can be used as reinforcement to increase the efficiency of filters with low performances or they can be used in combination with meso- and micro-porous structures to design filters with high selectivity towards specific contaminants.

An interesting example of filter systems reinforced with cellulose nanofibers is reported by Fukuzumi and co-workers, who produced polylactic acid (PLA) filters reinforced with a layer of TOCNF in order to decrease the oxygen permeability of PLA filters [93]. The presence of an additional layer of cellulose nanofibers, which have excellent transparency, flexibility, and tensile strength properties, led to a drastic reduction in the oxygen permeability of the filters from a value of 746 to a value of 1 mL m^−2^ day^−1^. Another example of cellulose-reinforced filters is the work of Nemoto et al. [13], previously reported for the synthesis of TOCNF spider-like aerogels, where commercially available HEPA filters were combined with TOCNF/water/TBA dispersions. These mixtures were prepared using 30% TBA and then freeze-dried, obtaining final TOCNF aerogel-containing filters, which showed superior filtration properties if compared to the starting HEPA filters.

TOCNF-COOH- and TOCNF-COONa coated on polyethylene terephthalate (PET) films were reported by Isogai et al. [81,82]. As for the self-standing layers previously described (Section 3.1.2), also in this case the gas permeability, particularly for H_2_, was observed.

**Figure 5:**
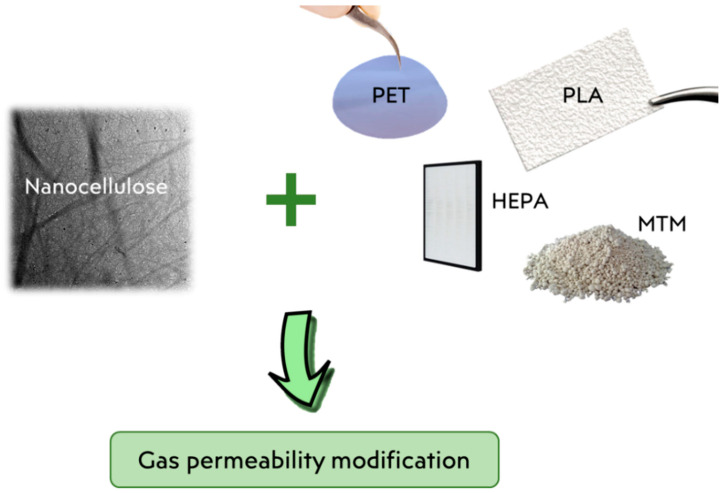
Representation of reinforced systems in which gas permeability is modified by the addition of nanocellulose.

Hybrid organic/inorganic nanocomposites can be also an innovative good alternative to produce filters materials which can arise specific properties. Isogai and co-workers synthesized a transparent nanocellulose/montmorillonite (MTM) composite film with increased morphological features caused by the homogeneous distribution of the MTM nanoplatelets in the TEMPO-oxidized cellulose matrix, mediated by the formation of weak interactions between the nano-layers [94]. This transparent and flexible new bio-based hybrid material exhibited a good oxygen permeability, which interestingly increases with the increment of MTM content. A graphical summary of the main reinforced systems discussed is given in Figure 5.

In addition to reinforced systems, combined systems are also an alternative solution for improving filter performance. An example was reported by Zhao and co-workers [95], who have prepared a porous CNF stringed HKUST-1 polyhedron membrane for air purification, combining the properties of CNF and the micro-porosity of the metal organic frameworks (MOF) HKUST-1 (or MOF-199) [96,97], resulting in a MOF-based nanofibrous thin layer. This filtrating system was able to efficiently adsorb formaldehyde, with an adsorption capacity of 47.71 mg g^−1^, and to block well PM_2.5_, while maintaining an air flow rate of 4.6 cm s^−1^ under 200 Pa, which is sufficient for the indoor activity.

As a subclass of MOFs built on imidazole ligands, zeolitic imidazolate frameworks (ZIFs) [98] could also be used to improve the surface area of cellulose-based filtering materials. As an example, Su et al. tested the possibility for the in situ growth of ZIF-8 nanocrystals onto cellulose microfibers [99]. A water: TBA dispersion of this hybrid material was then freeze-dried to obtain air filters with a spider web-like morphology. The successful incorporation of ZIF nanocrystals was confirmed by a remarkable increase in the BET surface area with respect to an unloaded sample (from 6.66 up to 620.80 m^2^/g). The introduction of ZIF-8 nanocrystals in the network allowed filtration efficiency to move from 99.5 to 99.9% against PM_0.3_ particles, while causing a three-fold increase in the pressure drop. As suggested by the authors, the formation of ZIF-8 agglomerates onto the fibers may clog the voids of the cellulosic net causing an increase in the air resistance.

**Figure 6:**
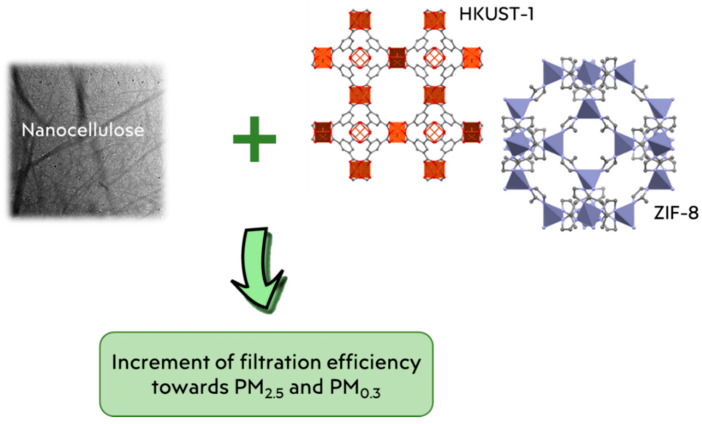
Representation of combined systems in which filtration efficiency is enhanced by the combination of the properties of nanocellulose and MOFs.

Combined filtering systems including MOFs are shown in Figure 6, while in Table 1 the main characteristics of the filtering systems reinforced and combined with nanocellulose are reported.

### 3.3. Cellulose Acetate-Based Systems

Among all the cellulose derivatives, the corresponding organic ester cellulose acetate (CA) has long been used for gas separation applications. A work reported by Paul and Puleo in 1989 demonstrated how the degree of acetylation is important to determine the transport characteristic of the most common gases, such as CO_2_ and CH_4_, within the fibrous structure. Indeed, the replacement of hydroxylic group with the bulkier acetyl groups induces a larger spacing between the chains, reducing the density of the final network, and consequently increasing the diffusion rates and the mobility of the gas molecules [100]. Later, the potentiality of cellulose acetate as building block for the production of gas separation membranes has been critically reviewed [101].

Considering the particles’ size as a function of their penetration, it is crucial to modulate the fiber diameter, the filter thickness, and its density in order to improve the filtration performance. Electrospinning is a technology mostly employed to generate fibers with altered morphological features. Chattopadhyay et al. exploited this technique to produce different types of cellulose acetate in order to investigate how the fiber diameters and the filter thickness influence the pressure drop and the particle penetration [102]. In particular, the authors observed that an increase of thickness above 40 mm has a negligible effect on the penetration grade of the NaCl aerosol particles while increasing the pressure drop value. Moreover, filters with micrometric diameters can delay the particle accumulation due to the larger inter-fiber distance. Overall, for electrospun acetate filters, the penetration of particles with size in the range of 40–270 nm can stand from 0.03% increasing up to 70%. To improve these beneficial features of cellulose acetate in gas adsorption, the design and the synthesis of novel CA-composites have been extensively investigated. An interesting example was reported by Chen et al. in 2014, who combined the large free volume of adamantane and the mechanical strength of cellulose acetate to produce novel composites with enhanced permeability coefficient for O_2_, N_2_, CO, CO_2_, and CH_4_ without losing any permeation selectivity [103].

An increase of gas permeability can be also attained forming a layered hybrid composite membrane of cellulose acetate and nanoporous silicate in low wt% as reported by Kim et al. [104]. This membrane resulted characterized by an exfoliated selective flakes/layer creating a system with higher CO_2_ permeability while preserving the CO_2_/CH_4_ selectivity. This behavior indicates a complex and tortuous diffusion mechanism of the gas molecules mediated by the porosity of the silicate, the interlayer space formed between the inorganic and organic components and the lightweight cellulose acetate matrix.

The control of nanofiller wt% to form mixed matrix membrane of CA may result in better gas permeation response when the nanofiller loading ratio increases. Moghadassi and co-workers revealed better performance for cellulose acetate-blend-multi walled carbon nanotubes mixed-matrix membranes (MMMs), especially in terms of CO_2_/CH_4_ separation [105]. They also evaluated the use of a second polymer blending in MMMs obtaining good results in terms of mechanical properties and membrane performance. The right ratio of cellulose acetate, capable to enhance the adsorption capacity of activated carbon nanofibers prepared by electrospinning and consequent thermal treatment of a polymeric blend consisting of CA and polyacrylonitrile, was studied by Yu et al. [106]. Once again, the combination of the high specific surface area, pore diameter, surface chemistry, and the micropores volume play a key role in the adsorption ability.

Cheng and coworkers reported the air filtration performance of electrospun cellulose acetate nanofiber membrane and its de-acetylated derivative. High filtration efficiency was detected, while a pressure drop value of 105 Pa was measured. Authors predict that by decreasing the fibers diameter and the pores size this pressure drop parameter can be reduced, thus considering the materials as good candidates for air filtration applications [107].

All the works reported in this current review paper described cellulose-based materials whose adsorption behavior results were controlled by mechanical and diffusive process mediated by the alteration of the morphological and physical features of the systems. However, chemical modification of the basic structure can be optimal to promote selective filtration efficiency against specific pollutants.

As the main component of cigarette filter the research about reaching higher degree of selectivity of CA for particulate smoke component is constantly expanding. Chemisorption may be the effectiveness process related the removal of certain toxic vapor constituents of the mainstream smoke [108]. Bourahla et al. was able to demonstrate that by coating the cellulose acetate fibers with branched polyethylenimine the aldehydic molecules can be attached on the surface of CA in form of hemiaminals [31]. In Table 1, cellulose acetate-based systems have been reported and synthetically characterized.

**Table 1:** Schematic summary of cellulose and nanocellulose-based materials and cellulose and nanocellulose-reinforced materials used as air filtering media.

Main Component of the Filtration System	Additional Component of the Filtration System	Type of Filter	Filtration Mechanism	Pollutant Targets	Performance Parameters Investigated	Reference
Cellulose	Glutaraldehyde and trimethylchlorosilane	Aerogel	Mechanical and chemical	Oil, organic solvents, lampblack	Sorption capacity, ∆*P*, η	[62]
TOCNF	-	Aerogel	Mechanical	PM_10_ dust	∆*P*, η	[65]
TOCNF	-	Aerogel	Mechanical	Particles (0.125–0.250 μm)	∆*P*, η	[13]
Cellulose	Surfactants	Aerogel	Mechanical	Air atmosphere	δ, % porosity, BET, permeability constant (K, µm^2^)	[66]
Wet-beaten softwood and hardwood kraft Pulp	-	Aerogel	Mechanical	NaCl aerosol particles (size 50–500 nm)	∆*P*, η	[68]
Kraft- or sulfite-pulp CNF	A-PAM	Aerogel	Mechanical	NaCl aerosol particles	∆*P*, η	[69]
Cellulose	Activated carbon	Aerogel	Mechanical and chemical	Benzene, toluene, ethylbenzene, and xylene, dust	BET, Adsorption isotherm	[70]
TOCNF	-	Film	Mechanical	O_2_	Young’s modulus	[81]
TOCN-COONa and TOCN-COOH	-	Film	Mechanical	O_2_, H_2_, N_2_, CO_2_	Tensile strength, thickness, ∆*P*, η	[82,83]
Bacterial cellulose	Magnetite nanoparticles	Membrane	Mechanical	Isopropanol, n-hexane	Equilibrium (saturation) adsorption capacity, q_i∞_ (g/g), and mean adsorption rate, v_i_ = q_i∞_/ τ _sat_ (g/g h)	[85]
Alkali/urea regenerated cellulose	Alkyl ketene dimer	Film	Mechanical	O_2_	Oxygen permeability, tensile strength, Young’s modulus, work of fracture	[84]
Self-fibrillating cellulose Fibers	-	Paper	Mechanical	O_2_	Young’s modulus, strain at break, optical transmittance	[88]
Softwood bleached kraft pulp	-	Paper	Mechanical	PM_0.3_	∆*P*, η	[89]
NBSK, NLF, and CTMP cellulose	VB and HW	Foams paper	Mechanical	NaCl aerosol particles	∆*P*, η	[91]
PLA-filter	TOCNF	Membrane	Mechanical	O_2_	Tensile strength, Young’s modulus, elongation, thermal expansion	[93]
PET-filter	TOCN-COONa and TOCN-COOH	Film	Mechanical	H_2_, N_2_, CO_2_	Tensile strength, thickness, ∆*P*, η	[82]
CNF	HKUST-1 (MOF)	Membrane	Mechanical and chemical	PM_2.5_ and formaldehyde	η, air flow rate	[95]
Cellulose	ZIF-8	Paper	Mechanical	PM_0.3_	∆*P*, η	[99]
TOC	MTM nanoplatelets	Film	Mechanical	O_2_	Young’s modulus, tensile strength, elongation, ∆*P*, η	[94]
Cellulose acetate	-	Electrospun filter	Mechanical	Diethyl hexyl sebacate aerosol particles, NaCl aerosol particles	Fiber diameters, thickness, solidity, ∆*P*, η	[102]
Cellulose acetate	Adamantane	Membrane	Mechanical	O_2_, N_2_, CH_4_, CO and CO_2_	Permeability coefficient, diffusion coefficient, solubility coefficient	[103]
Cellulose acetate	Nanoporous silicate	Membrane	Mechanical	CO_2_, CH_4_	Thickness, ∆*P*, interlayer space	[104]
Cellulose acetate	Multi-walled carbon nanotubes (MMMs)	Membrane	Mechanical	O_2_, N_2_, CO_2_, CH_4_, He	∆*P*, flux, membrane thickness, membrane area	[105]
Cellulose acetate	Polyacrylonitrile	Electrospun filter	Mechanical	Toluene	Total pores volume, specific surface area, average pores diameters, breakthrough time and capacity	[106]
Cellulose acetate	Branched polyethylenimine	-	Mechanical and chemical	-	-	[31]

## 4. Conclusions

The aim of this review was to elucidate how cellulose can be employed in processing filtering materials highlighting both the potentialities and the beneficial modifications.

By considering the revised works herein discussed, some main conclusions could be drawn. First, it emerges clear how regulating the physical and chemical properties of the cellulose fibers should be considered a critical issue in order to design and develop efficient air-filters, with nanosized fibers which determine in several circumstances a significant improvement. In this context, electrospinning of nano-cellulose opens the way to a wider range of hierarchical solutions fruitful to enhance filtration performances. The use of emerging technologies, such as 3D printing of (eco)safe filters could more and more drive the morphology design for an efficient filtration. Moreover, the formulation of composites, where cellulose can be the matrix or the reinforce component, in most cases should be considered as the preferred route to achieve good gas-filtration properties. In addition, the use of TBA as the solvent media in processing aerogel systems can be an example to keep in mind whether microporous open structures need to be obtained for filtration of micro-sized volatile particles, as it provides a good compromise between the necessity to be effective in the removal of contaminants and, at mean time, the need to prevent an undesired increase of pressure drop.

Up to now, there have not been many examples of cellulose-based materials with specific gas adsorption properties, and this aspect should be further investigated. Moreover, the request for advanced solutions for air sanitization, made even more stringent by the current pandemic situation, makes the doping of cellulose-based materials with antimicrobial/antiviral agents a topic of great interest, which deserves much attention from scientific community in the near future [109,110]. Thus, we wish that this critical review might represent a starting point and a guideline for all those groups which aim to enter the research world of efficient eco-friendly and sustainable cellulose-based air-filters.

## Data Availability

Not applicable.
